# Value of stent boost imaging in decision making after coronary stenting

**DOI:** 10.1007/s10554-023-02961-4

**Published:** 2023-10-17

**Authors:** Hossam M. Mansour, Ahmed M. Mohamed, Soliman G. Ibrahim, Ayman M. Ibrahim, Ramadan G. Mohamed

**Affiliations:** 1 Department of Cardiology, Faculty of Medicine, Aswan University, Aswan, 81528 Egypt; 2 Department of Cardiology, Faculty of Medicine, Cairo University, Cairo, Egypt

**Keywords:** PCI, Stent Boost Imaging, IVUS, DES

## Abstract

**Background:** Several studies reported the comparability of digital stent enhancement techniques (including stent boost imaging) in detecting suboptimal results of coronary stenting with Intra Vascular Ultrasound and optical coherence tomography. **Aims:** to assess results of stent deployment and determine the incidence of suboptimal results requiring changing final decision using stent boost imaging. **Methods:** This cross-sectional study included 120 patients eligible for PCI were recruited during a period of one year (January 2021 to 2022) using DES. **Results:** Suboptimal results were found in 38% of the PCI cases with stents (angiography guided). Importantly it was found that improper lesion preparation in our practice could not help improving stent optimization. Also, angiography guided PCI has significant incidence of suboptimal results. Digital stent enhancement techniques like stent boost have significant and important value in better decision making. After adjusting for age and sex, six factors were identified as independent predictors for final decision change (stent length, LAD/RCA affection, proximal segment affection, calcification, and optical coherence tomography. **Conclusion:** This study has confirmed the utility of stent boost for the optimization of PCI in daily practice. Stent Boost is a simple and costless technique that provides an accurate assessment of a deployed stent without extending the procedure time and without more risk. It appears to be useful for the immediate evaluation of stent expansion and optimization of PCI by additional post-dilatation, when appropriate. Future studies are needed to determine whether Stent Boost data will correlate with adverse long-term clinical outcomes in patients undergoing PCI.

## Introduction

The first percutaneous transluminal coronary angioplasty (PTCA) was performed by the German cardiologist Andreas Gruentzig on September 16, 1977. After that, the use of intracoronary stents was identified as a method to treat some complications due to (PTCA) [1]. It was realized that restenosis rates were significantly lower in individuals who received an intracoronary stent when compared to those underwent balloon angioplasty [2, 3].

Further, it was apparent that these stents led to troubling phenomena of in-stent restenosis (ISR) and stent thrombosis, which required repeat revascularization, associated with increased morbidity and mortality, and posed a therapeutic challenge. Continued efforts were devoted for improvement of stent technology for optimal stent complications therapy. These included design and alloy modification, reducing strut thickness, and adding a polymer to elute an antiproliferative drug, drug-eluting stents (DES) that significantly reduced the occurrence of exuberant neointimal proliferation [4, 5]. However, despite the wide use and experience gained with novel stent technologies and implantation techniques, the rates of ISR are still relatively high and stent thrombosis still occur significantly [6–8].

Major aetiologies for ISR and stent thrombosis have been traditionally classified and characterized: operator or technique dependent (including stent under-sizing, incomplete lesion coverage, stent under expansion, and malposition), design properties of stents (that may lead to recoil, stent fractures, and altering increase in shear stress), patient and biologically related conditions [9].

**Fig. 1:**
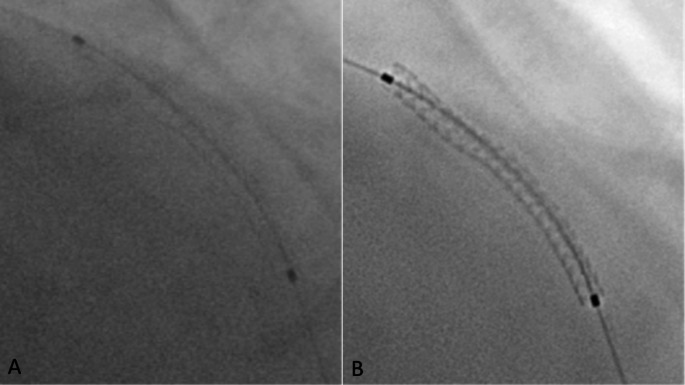
How coronary stent could be visualized using basal angiography **(A)** versus Stent boost image **(B)**

**Fig. 2:**
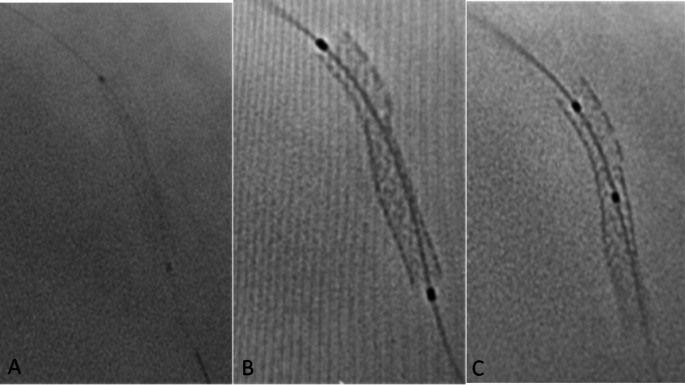
Stent boost image detected focal stent non-expansion that needed further optimization. **(A**: basic angiographic image, **B** & **C**: stent boost images before and after post dilation.)

**Fig. 3:**
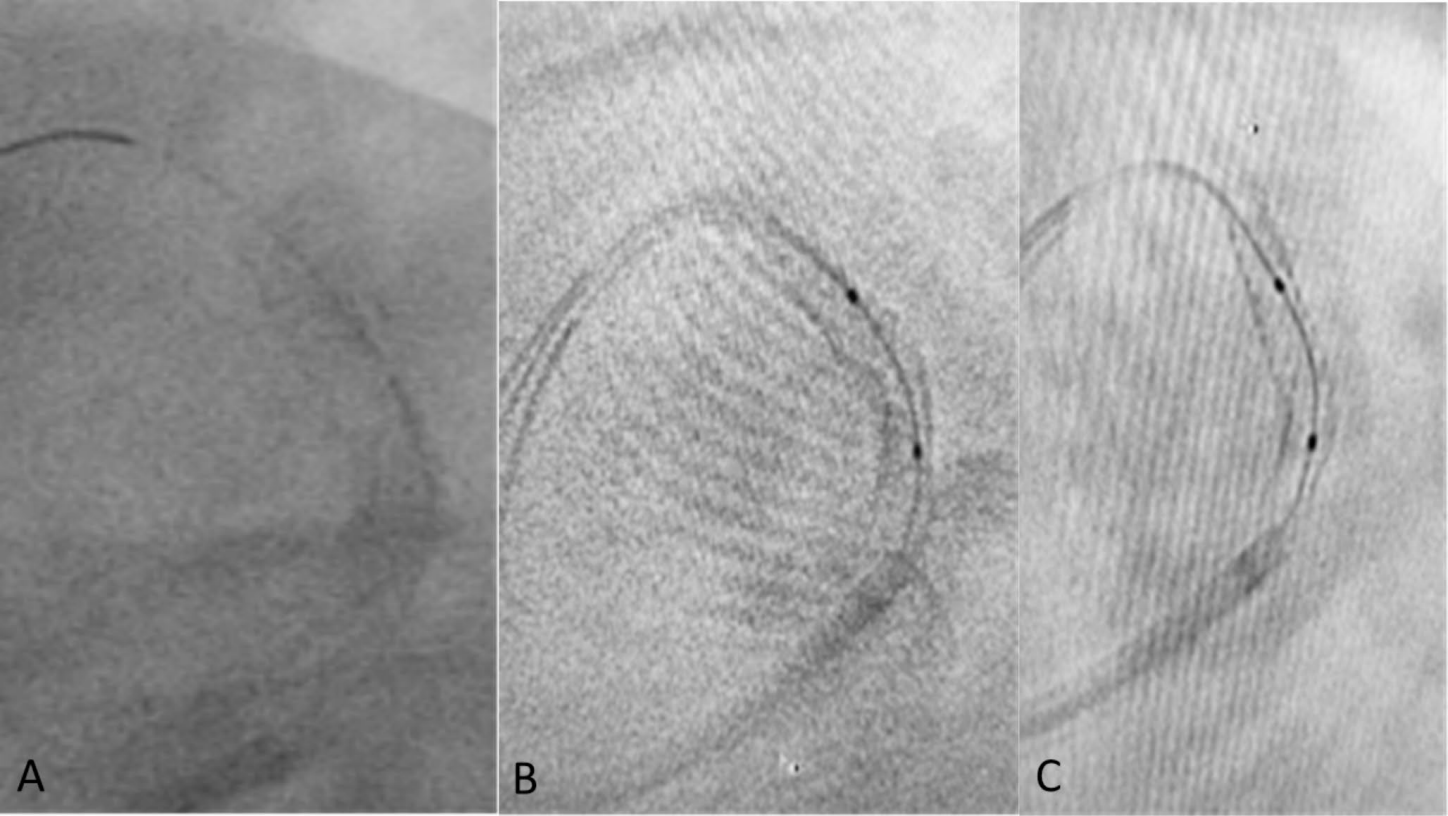
Stent mal apposition (due to calcium) could be seen after stent boost imaging, so, stent optimization was done

Mechanical and implantation factors such as stent under-sizing, stent fracture or inadequate deployment are the most important and controllable factors responsible for acute and chronic complications as evidenced by various studies [10–12]. These conditions are not always easily assessable on basic angiographic images that also underestimate stent under-expansion. Intra Vascular Ultrasound (IVUS) can be used to give a conclusive answer on the presence of malposition and under-expansion, but it adds time and costs to the procedure and requires training. Additionally, while uncommon, complications have been reported [13, 14].

Stent Boost (SB) imaging has been developed by Philips Medical Systems based upon techniques that enhance the radiologic edge of the stent, improving visualization of the stent struts. It is comparable to IVUS, can be used to guide stent optimization and to assess stent characteristics, The methodology is extremely safe, user-friendly, cheap, and does not significantly increase radiation exposure or procedural time, it requires only an extra cine-run of a few (3–4) seconds [15, 16].

The specificity of SB image for adequate stent deployment is high (100% when assessed by IVUS, and 96% when assessed by optical coherence tomography (OCT)), so it could be used as the first line for monitoring just after stent implantation in centres where IVUS is not routinely used. [17, 18]. In our practice we do not use IVUS frequently for assessing the results of stent deployment, which is a major requirement that should be fulfilled. So, during this work we assessed results of stent deployment and determined the incidence of suboptimal results requiring changing final decision using SB imaging.

## Patient and methods

This cross-sectional study was conducted at Aswan university hospital Cath. Lab, patients eligible for PCI were recruited during a period of one year (January 2021 to 2022) using DES. Using the epi-info-7 software for calculation of sample size with the following assumptions: cross sectional study, 80% power, 95% significance level: 20% of outcome in unexposed group, 2.5 risk ratio, 2.2% outcome in exposed group. The required sample size was 118 cases [19].

After completing the routine angiography guided PCI, the result of stent deployment was assessed by stent boost imaging (Fig. 1). After implantation of the stent, an additional cine run of a few seconds is made with the deflated balloon still in place without contrast. Taking the balloon markers as landmarks, all individual images in subsequent cine runs are superimposed by translation, dilatation, and rotation. The SB software automatically performs a stent edge enhancement, and thereafter quantitative stent measurements are taken. Stent diameter is calculated with manual tracing of the longitudinal stent edges in the enhanced images using the guiding catheter as a reference. The minimum diameters of the stent and stent edge diameters as the reference of the stent will be determined automatically using the SB software.

A stent was optimally deployed and expanded if all the following criteria were fulfilled using SB imaging [15]: (A) No sign of focal inadequate expansion, as no protrusion of the stent strut and no disappearance of continuity of stent struts (Figs. 2 and 3). (B) Stent minimum diameter > 70% of reference diameter (diameter of the vessel at the site of lesion according to QCA assessment). (C) Stent minimum diameter > 2.0 mm. The symmetry index of the stent is to be calculated using SB image. The symmetry index was calculated as the minimum diameter/maximum diameter, it should be > 0.7 [20]. Number of cases in whom decision has been changed as they need further optimization after SB imaging will be calculated as % of total cases.

### Statistical analysis

Data were verified, coded by the researcher, and analysed using IBM-SPSS 24.0 (IBM-SPSS Inc., Chicago, IL, USA) [21]. Descriptive statistics: Means, standard deviations, medians, ranges, frequency, and percentages were calculated. Test of significances: Chi square and Fisher Exact tests were used to compare the difference in distribution of frequencies among different groups as appropriate. Shapiro-Wilk test will be used to test for data normality. Student t-test and Mann-Whitney U test were calculated to test the mean differences in continuous variables between groups (parametric and non-parametric). The clinical and demographic factors with proven statistical significance were further included in the multivariable logistic regression models to explore the main factors affecting final decision change. Significant p value was considered when it is < 0.05.

### Ethical consideration

IRB approval was obtained from the Medical Ethic Committee, Faculty of Medicine, Aswan University (Asw.U./243/5/18). The study was carried out in accordance with the Helsinki Declaration guidelines [22] and in line with STROBE checklist for research ethics. The title and objectives of the study were explained to them to ensure their cooperation. A written informed consent was obtained from the patient before the participation in the study. All collected data was confidential and was used for the purpose of scientific research only. Every research participant had the complete right and freedom to withdraw at any time from the study without any consequences on the medical service provided.

## Results

This cross-sectional study included 120 patients with IHD eligible for elective PCI using DES at Aswan University Hospital catheterization laboratory collected during a period of one year.

### Baseline characteristics of the studied sample

The basic demographic and clinical data of the studied cohort were shown in Table 1. The patients’ age ranged between 35 and 80 years. Male represented about three-quarters and smokers represented about two-thirds of the sample. For the prevalence of comorbidity, about 58% of the sample had DM, about 57% had HTN, about 13% had chronic kidney disease, about 6% had peripheral vascular disease/stroke/ obstructive sleep apnea/hypothyroidism.

**Table 1:** Baseline Characteristics of the studied Cohort

Variable	Category	n = 120
**Age/years**	• **Mean ± SD**	57.44 ± 10.3
	• **Median (Range)**	58 (35–80)
**Sex**	• **Female**	23 (26.7%)
	• **Male**	88 (73.3%)
**Smoker**	• **No**	46 (38.3%)
	• **Yes**	74 (61.7%)
**DM**	• **No**	51 (42.5%)
	• **Yes**	69 (57.5%)
**HTN**	• **No**	52 (43.3%)
	• **Yes**	68 (56.7%)
**Other Comorbidities**	• **CKD**	16 (13.3%)
	• **Hypothyroidism**	1 (0.8%)
	• **OSA**	2 (1.7%)
	• **PVD**	5 (4.2%)
	• **Stroke**	5 (4.2%)

### Distribution of stents used among studied cohort

**Fig. 4:**
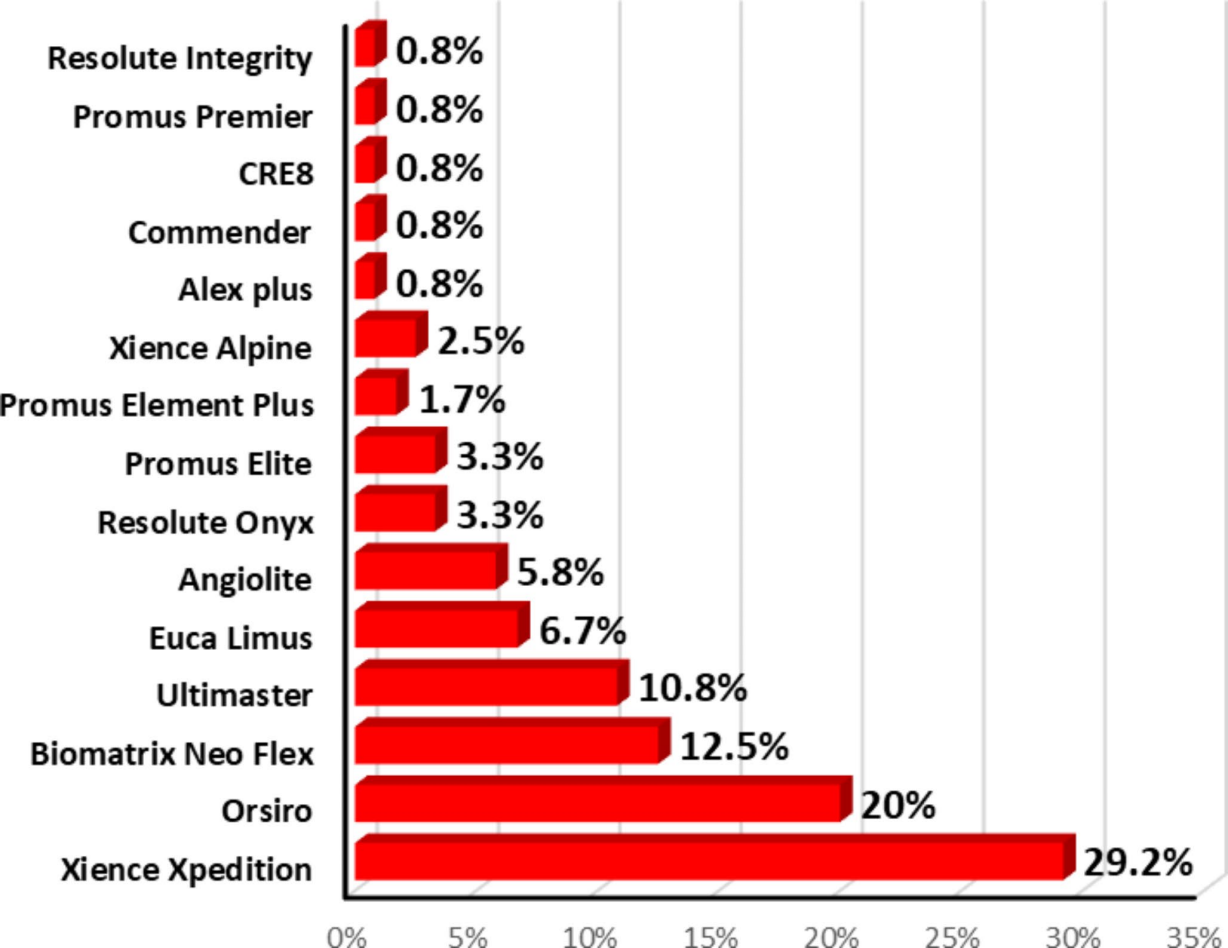
Distribution of the studied Sample according to Stent Type

Distribution of the stent type was as follows: about one-third used Xience Xpedition, 20% used Orsiro, 12.5% used Biomatrix Neo Flex, about 11% used Ultimaster, about 7% used Euca Limus, about 6% used Angiolite, about 3% used Resolute Onyx/Promus Elite, 2.5% used Xience Alpine, 1.7% used Promus Element Plus and 0.8% used Alex plus/Commender/CRE8/Promus Premier/Resolute Integrity (Fig. 4).

### Characteristics of the studied sample according to the targeted vessels

Table 2 showed the characteristics of the studied sample regarding vessels targeted. vessels were matched for symmetrical and asymmetrical stents. For the distribution of the affected vessels, LAD was the target vessel in about 59%, RCA was the target vessel in 22.5%, LCX was the target vessel about 14%, and in 5% OM/Diagonal vessels were targets. Regarding the affected segment, about 55% had mid-segment affection, about one-third had proximal segment affection and about 12% had distal segment affection. Moreover, the mean reference vessel diameter was 3.2 ± 0.4 mm2 with a median of 3.2 and a range of 2.2–4.3 mm2.

**Table 2:** Clinical Characteristics of the studied Cases according to target vessel

Variable	Category	n = 120
Affected Vessel	• LAD	71 (59.2%)
	• RCA	27 (22.5%)
	• **LCX**	**17 (14.1%)**
	• Others*	**5 (4.2%)**
**Affected Segment**	• **Distal**	**14 (11.7%)**
	• **Mid**	**66 (55%)**
	• **Proximal**	**40 (33.3%)**
**Reference Vessel Diameter**	• **Mean ± SD**	**3.21 ± 0.4**
	• **Median (Range)**	**3.2 (2.2–4.3)**

*Others = OM, Diagonal

### Imaging findings of the studied sample

For the Imaging results, 40% required pre-dilatation, about one-quarter seemed to have angiographically high calcific burden, about 4% had CTO and only 0.8% (n = 1) had bifurcation lesion. Regarding the inflation data, the mean stent inflation pressure was 16.3 ± 2.1 with a median of 16 and a range of 12–22. Also, the mean stent inflation seconds was 19.3 ± 4.2 with a median of 20 and a range of 5–30. Number of cases that required post-dilatation of the stent were 46 (38.3%) (Table 3).

**Table 3:** Imaging Findings of the studied Cases

Variable		Category	n = 120
**Imaging Findings**		• **Calcification**	31 (25.8%)
		• **CTO**	5 (4.2%)
		• **Bifurcation**	1 (0.8%)
		• **Pre-dilatation**	48 (40%)
**Stent Inflation Pressure**		• **Mean ± SD**	16.29 ± 2.1
		• **Median (Range)**	16 (12–22)
**Pre-Dilatation**		• **Yes**	48 (40%)
**Post-dilatation after Angiographic Assessment**			
	• **Yes**		46 (38.3%)

### Outcome results of the studied sample

**Fig. 5:**
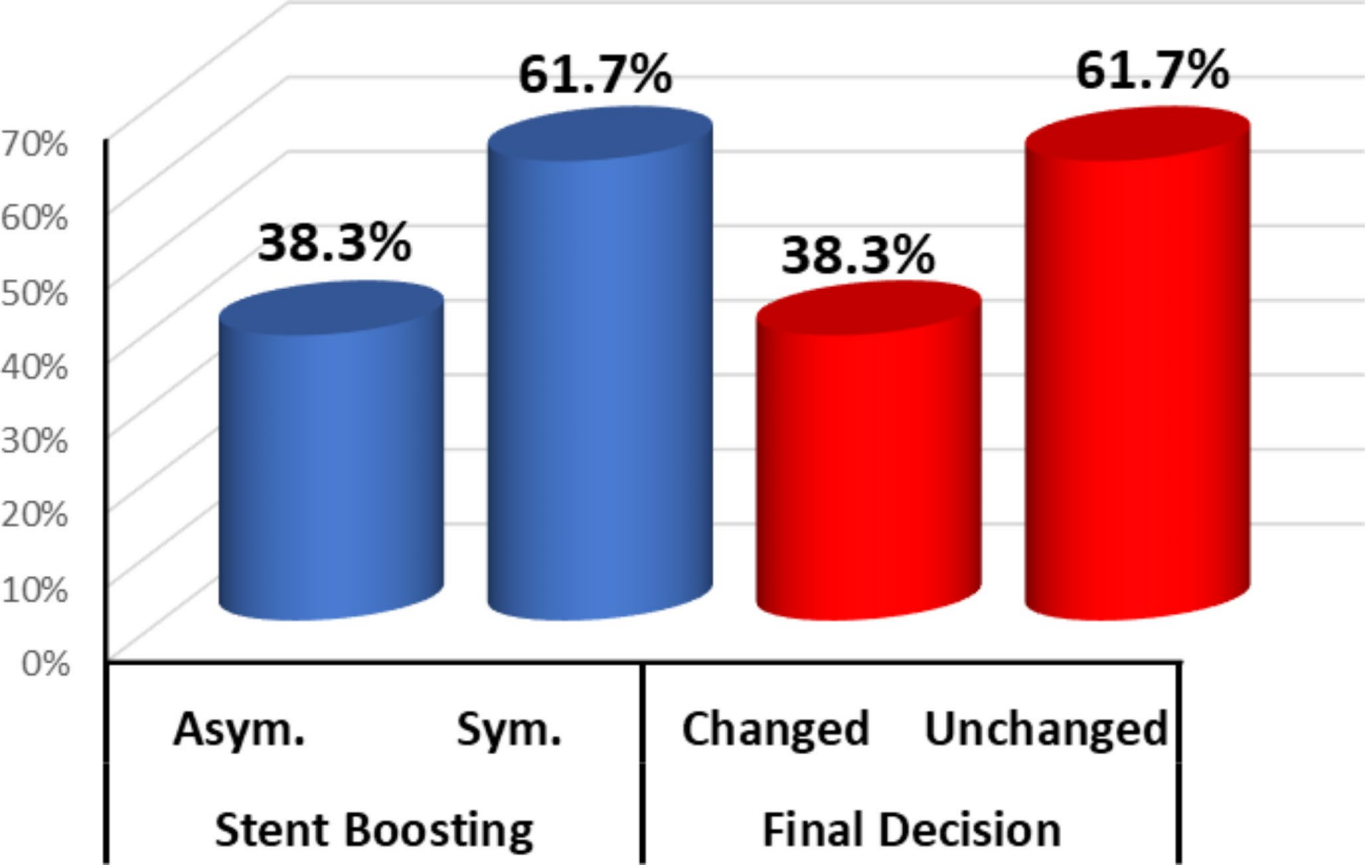
Outcome Findings among the studied Groups

After reaching the angiographic optimization of the stent and decided as to have visually good result, stent boost images were obtained that revealed the occurrence of suboptimal results (asymmetrical stents) in 38% of stents deployed, accordingly final decision was changed for further optimization needed (Fig. 5).

### Effect of stent Length/SB imaging on the final decision

As demonstrated in Table 4, there was significant relationship between stent length and final decision (p = 0.046) i.e., increase in stent length was more evident in those with changed decision (suboptimal results) (13%, 39% and 48%) compared with unchanged decision (24%, 42% and 34%).

**Table 4:** Effect of Stent Length/SB Imaging on the Final Decision

	Final Decision		P-value*
	Changed (n = 46)	Unchanged (n = 74)	
**Stent Length**			
• **< 20 mm**	**6 (13.1%)**	**18 (24.3%)**	**= 0.046**
• **20–30 mm**	**18 (39.1%)**	**31 (41.9%)**	
• **> 30 mm**	**22 (47.8%)**	**25 (33.8%)**	
**Stent Boost Image Findings**			
• **Asymmetrical**	**46 (100%)**	**0 (0%)**	**< 0.001**
• **Symmetrical**	**0 (0%)**	**74 (100%)**	
**Pre-dilatation**			
• **No**	**27 (58.7%)**	**45 (60.8%)**	= 0.818
• **Yes**	**19 (41.3%)**	**29 (39.2%)**	
**Post-dilatation**			
• **No**	**35 (76.1%)**	**69 (93.2%)**	**= 0.007**
• **Yes**	**11 (23.9%)**	**5 (6.8%)**	

*Chi-square test was used to compare differences in frequency between groups

**Table 5:** Independent Predictors of Final Decision Change: Multivariable Logistic Regression Model

	OR (95% CI) *	P-value
• **Age/years**	1.006 (0.971–1.043)	= 0.726
• **Sex (Male)**	0.648 (0.286–1.469)	= 0.289
• **Smoker**	0.557 (0.267–1.182)	= 0.127
• **Stent Length (mm)**	**1.031 (1.001–1.078)**	**= 0.044**
✓ **< 20 mm**	**1**	**= 0.007**
✓ **20–30 mm**	**0.348 (0.117–0.801)**	**= 0.048**
✓ **> 30 mm**	**0.606 (0.198–0.915)**	**= 0.032**
• **Stent Diameter (mm)**	1.489 (0.584–3.769)	= 0.404
• **Affected Vessel**		
✓ **LAD**	**0.890 (0.423–0.873)**	**= 0.038**
✓ **RCA**	**1.958 (1.002–4.656)**	**= 0.046**
✓ **LCX**	0.605 (0.198–1.845)	= 0.377
✓ **Others**	0.375 (0.041–3.463)	= 0.387
• **Affected Segment**		
✓ **Proximal**	**1.232 (1.004–2.669)**	**= 0.027**
✓ **Mid**	0.917 (0.432–1.940)	= 0.821
✓ **Distal**	0.604 (0.112–3.251)	= 0.558
• **Calcification**	**0.475 (0.200–0.763)**	**= 0.047**
• **CTO**	**1.423 (1.021–4.101)**	**= 0.034**
• **Pre-dilatation**	1.092 (0.516–2.321)	= 0.818
• **Post-dilatation**	**0.241 (0.078–0.746)**	**= 0.014**
• **Inflation**		
✓ **Pressure**	1.037 (0.865–1.244)	= 0.692
✓ **Seconds**	1.001 (0.916–1.093)	= 0.991
✓ **Number**	1.368 (0.619–3.022)	= 0.439

**OR = Odds Ratio; CI, Confidence Interval**

### Relationship between pre-dilatation, post-dilatation, and final decision change

In this study, there was an insignificant relationship between final decision and pre-dilatation (p = 0.818). It was found that among cases that underwent post-dilation according to the result of basic visual angiographic assessment, there was still a higher incidence of suboptimal results and need for further optimization (changing final decision) when assessed by stent boost image (p = 0.014) in comparison with other cases.; quarter of cases (23.9%) with final decision change had post-dilatation vs. only 6.8% (n = 5) of unchanged decision (p = 0.007).

### Predictors of final decision change

After adjusting for age and sex, six factors were included (stent length, LAD/RCA affection, proximal segment affection, calcification, cases required post-dilation depending on basic angiographic assessment, inflation pressure and duration and CTO). It was found that with a one-mm increase in the stent length there was a 3% increase in the chance of change of the final decision due to suboptimal results. Regarding vessel affected, patients with LAD affection had 11% reduction in the liability of change of the final decision. Contrarily, patients with RCA affection had double the risk of change of the final decision. Likely, those with proximal segment affection had 23% likelihood of having change of the final decision.

Moreover, presence of calcification was associated with 53% decrease in the likelihood of having change of the final decision. On the other hand, the presence of CTO was associated with 42% increase in the probability of having change of the final decision. Among cases that underwent post-dilation according to the result of basic visual angiographic assessment, there was still a higher incidence of suboptimal results and need for further optimization when assessed by stent boost image in comparison with other cases There was no significant relationship between inflation pressure of the stent or duration of inflation in seconds.

## Discussion

Myocardial revascularization represents the most frequently performed therapeutic intervention worldwide [23, 24]. The need for repeat revascularization has a significant impact on quality of life and exposes patients to risks intrinsically related to repeat hospitalizations and invasive procedures [25].

Advances in stent technology have improved PCI results; however, acute (stent thrombosis) and late (in-stent restenosis/thrombosis) complications still occur [26]. In our practice, PCI results are just assessed visually by basic angiography in most daily practice cases. This work aimed to study the value of stent boost imaging in changing the final decision after angiography guided PCI for better optimization.

In this study, value of stent boost imaging in detecting suboptimal results and changing final decision is significant, this was in agreement with the results of study conducted by Blicq et al., [27] that reported the significant value of SB imaging in decision making for better stent optimization in angiography guided PCI. In the current study, final decision was changed for further optimization due to suboptimal results after being assessed further by using SB imaging in 38% of cases. This was lower in Blicq et al. (18%) indicating some differences in the PCI practice. This difference mostly attributed to the larger percentage of cases needed pre-dilation that was not ideally performed (using small sized PTCA semi-compliant balloons, no use of 1:1 balloon to vessel size, no use of NC balloons; noting that 40% of our study cases needed pre-dilatation). Similarly, the current results were consistent with Yuanyuan Duan et al. [18] as they found suboptimal results in 24% of STEMI cases (all study cases were acute MI, indicating more soft lesions that were stented). Also, Kang et al. [28] found that 42% of cases during their study had stent under expansion by IVUS.

Furthermore, there was a significant association between stent length and the possibility of suboptimal results changing final decision (especially for stent length exceeding 30 mm). This agreed with other studies that showed a significant relationship between stent length and outcomes including both ISR and stent thrombosis [28–30]. In this study, it was found that with one-mm increase in the stent length there was 3% (OR = 1.031, 95% CI; 1.001–1.078) increase the chance of change of the final decision due to suboptimal results. Contrarily, there was no significant correlation between stent diameter and possibility of either end results, also no significant effects of inflation pressure or inflation seconds. Proximal segment lesions treated with PCI had significantly higher possibility of changing final decision for further optimization other than PCI to lesions at mid or distal parts. This may be explained by the improper sizing of the stent at proximal segments depending only on angiographic assessment. Vessel under sizing when using angiographic assessment has been recently reported in a recent study done by Goel et al., [31].

Also, more broad segments (especially LM) were found to have higher possibility of suboptimal results and the need for further optimizations in the study done by Blicq et al., [27]. Regarding vessels affected, angiography guided PCI to RCA was found to have significantly higher possibility of changing final decision for further optimization needed according to stent boost result, this was insignificant for the other vessels. Those with calcifications were significantly less liable for changing final decision, this may be because of the presences of calcification itself, this is the cause that the operator is more careful about best stent optimization could be reached within the calcific lesions with best lesion preparation & post dilation more frequently done as a routine step, this was also in agreement with the results found by Blicq et al. [27] in their work, there was no impact of the presence of calcification on the final decision after stent boost evaluation.

In accordance with a study conducted by Nailing et al. [32] who found that the incidence of suboptimal results of angiography guided CTO cases was significantly higher when compared to IVUS guided CTO PCI, i.e., cases of CTO PCI have significantly higher possibility of suboptimal results and further stent optimization. Also, the current results found an insignificant relationship was reported between pre-dilatation and decision change, this was in contrast with Park et al. [30] who found that pre-dilatation is associated with better outcomes.

Among cases that underwent post-dilation according to the result of basic visual angiographic assessment, there was still a higher incidence of suboptimal results and need for further optimization (changing final decision) when assessed by stent boost image (p = 0.014) in comparison with other cases. This was contradictory to Park et al. [30] who found post-dilatation was significantly associated with better outcomes. These two observations may be attributed to missing an ideally performing pre-dilatation, lesion preparation and post-dilatation in our study practice (that was done routinely in Pak et al. study, was guided by intracoronary imaging before stent deployment, and using more appropriately sized NC Balloons for pre- and post-dilatation). Our findings regarding post-dilatation came in accordance with results of Lee et al. 2022 study who found that postintervention quantitative coronary angiography–based minimum lumen diameter was not different between the angiography guidance with post-dilation versus the angiography guidance without post-dilation group and concluded that in patients undergoing long drug-eluting stent implantation, IVUS-guided post-dilation was associated with improved long-term clinical outcomes, unlike angiography-guided post-dilation [33].

To our knowledge, the current study was one of few studies estimating the value of the DSE (Digital stent enhancement namely SB) for better detection of suboptimal results in daily practice. These results confirmed the significance of using this SB imaging technique and its importance in optimizing acute PCI results and hence better long-term outcomes. Knowing that the gold standard methods such as IVUS and OCT are performed in < 10% of routine PCI [27], this makes the value of DSE as stent boost more and more important being simple, cheap, more available tool in a large proportion of cath. Labs and without procedural risk DSE as stent boost should be recognized as an essential step of routine PCI practice and as a quality parameter of coronary intervention using coronary stents. This may help in significant reduction of incidence of ISR and stent thrombosis.

### Study limitations

This study encountered several limitations: it is a single center study, little data are available in Egypt about the use of enhanced stent imaging techniques, so our results are not compared or correlated with data about the same population and practice, it included all consecutive PCIs performed in the center during the study period, whatever the indication, the analysis of the data did not interfere with the PCI process, in all cases, the decision to post-dilate and the choice of the balloon for post-dilatation were at the discretion of the operator, in this study, SB images were acquired in only one view selected by the operator to minimize foreshortening and interference from other radio-dense objects.

## Conclusion

In conclusion, the study has confirmed the utility of SB for the optimization of PCI in daily practice. SB is a simple and costless technique that provides an accurate assessment of a deployed stent without extending the procedure time and without more risk. It appears to be useful for the immediate evaluation of stent expansion and optimization of PCI by additional post-dilatation, when appropriate. Future studies are needed to determine whether SB data will correlate with adverse long-term clinical outcomes in patients undergoing PCI.

## Data Availability

Data is available upon request from the corresponding author.
